# The White Matter Functional Abnormalities in Patients with Transient Ischemic Attack: A Reinforcement Learning Approach

**DOI:** 10.1155/2022/1478048

**Published:** 2022-10-17

**Authors:** Huibin Ma, Zhou Xie, Lina Huang, Yanyan Gao, Linlin Zhan, Su Hu, Jiaxi Zhang, Qingguo Ding

**Affiliations:** ^1^School of Information and Electronics Technology, Jiamusi University, Jiamusi, China; ^2^Integrated Medical School, Jiamusi University, Jiamusi, China; ^3^Department of Radiology, Changshu No.2 People's Hospital, The Affiliated Changshu Hospital of Xuzhou Medical University, Changshu, Jiangsu, China; ^4^School of Teacher Education, Zhejiang Normal University, Jinhua, China; ^5^Key Laboratory of Intelligent Education Technology and Application of Zhejiang Province, Zhejiang Normal University, Jinhua, China; ^6^Faculty of Western Languages, Heilongjiang University, Heilongjiang 150080, China

## Abstract

**Background:**

Transient ischemic attack (TIA) is a known risk factor for stroke. Abnormal alterations in the low-frequency range of the gray matter (GM) of the brain have been studied in patients with TIA. However, whether there are abnormal neural activities in the low-frequency range of the white matter (WM) in patients with TIA remains unknown. The current study applied two resting-state metrics to explore functional abnormalities in the low-frequency range of WM in patients with TIA. Furthermore, a reinforcement learning method was used to investigate whether altered WM function could be a diagnostic indicator of TIA.

**Methods:**

We enrolled 48 patients with TIA and 41 age- and sex-matched healthy controls (HCs). Resting-state functional magnetic resonance imaging (rs-fMRI) and clinical/physiological/biochemical data were collected from each participant. We compared the group differences between patients with TIA and HCs in the low-frequency range of WM using two resting-state metrics: amplitude of low-frequency fluctuation (ALFF) and fractional ALFF (fALFF). The altered ALFF and fALFF values were defined as features of the reinforcement learning method involving a *Q*-learning algorithm.

**Results:**

Compared with HCs, patients with TIA showed decreased ALFF in the right cingulate gyrus/right superior longitudinal fasciculus/left superior corona radiata and decreased fALFF in the right cerebral peduncle/right cingulate gyrus/middle cerebellar peduncle. Based on these two rs-fMRI metrics, an optimal *Q*-learning model was obtained with an accuracy of 82.02%, sensitivity of 85.42%, specificity of 78.05%, precision of 82.00%, and area under the curve (AUC) of 0.87.

**Conclusion:**

The present study revealed abnormal WM functional alterations in the low-frequency range in patients with TIA. These results support the role of WM functional neural activity as a potential neuromarker in classifying patients with TIA and offer novel insights into the underlying mechanisms in patients with TIA from the perspective of WM function.

## 1. Introduction

Stroke is one of the leading causes of morbidity, mortality, and loss of function worldwide [1, 2]. The increasing prevalence of stroke places a tremendous economic burden on individuals and society [3]. Transient ischemic attack (TIA), also known as “ministroke,” is a serious, reversible, temporary neurological condition caused by focal cerebral nervous system hypoperfusion [4]. It is acknowledged that TIA is a continuum with stoke in the presentation of acute cerebrovascular events [5]. Therefore, precise diagnosis and effective treatment of TIA are paramount to reducing the risk of subsequent stroke [6–8]. To promote targeted treatment and precise identification of TIA, advanced imaging techniques have been applied to explore the underlying mechanism.

In recent years, resting-state functional magnetic resonance imaging (rs-fMRI) has been considered a promising imaging technique for studying gray matter (GM) alterations based on blood oxygen level-dependent (BOLD) signals [9–13]. However, the signals in white matter (WM) were often neglected as noise, because it was previously thought that WM could not generate BOLD signals due to few postsynaptic potentials [14–17]. Therefore, previous studies of WM in patients with TIA were mainly structural. For instance, structural abnormalities have been found in the superior longitudinal fasciculus in the WM, implying impaired sensorimotor function in patients with TIA [18, 19]. Increasing evidence indicates the existence of functional information in WM that can be reliably detected by BOLD fMRI [20–24], which might open new avenues for studying WM in health and disease. By combining fMRI and dynamic positron emission tomography (PET), BOLD fluctuations in WM have been found to correlate with neural activity through local variations in glucose metabolism, suggesting a possible physiological basis for WM function [25]. Particularly, it has been demonstrated in healthy participants that spontaneous low-frequency BOLD fluctuations in WM can be robustly detected and reflect specific neural activities [26, 27]. During the resting state, Peer et al. [28] applied the Fourier transform of WM functional network signals obtained from healthy participants, and greater neural activity at low frequencies was found to exist in WM networks. A similar feature of neural activity at low-frequency bands in WM has been detected in several neurological or mental diseases, such as schizophrenia [29, 30] and epilepsy [31]. Besides, the WM function estimated by low-frequency BOLD signals can also be modulated by different tasks, suggesting the possibility of estimating the dynamic function of WM fiber bundles using low-frequency BOLD fluctuations [27, 32, 33]. Considered together, these studies provide strong evidence that meaningful signals exist in WM and that low-frequency fluctuations in WM could be effectively detected by BOLD fMRI. Nevertheless, it remains unknown whether there are abnormal functional alterations in low-frequency bands of the WM in patients with TIA. Thus, we expected that unveiling the low-frequency BOLD fluctuation characteristics in the WM of patients with TIA may provide additional information about WM dysfunction in TIA and help better understand the underlying pathological mechanisms of TIA.

Two effective resting-state methods have been raised to characterize the features of low-frequency BOLD fluctuations: amplitude of low-frequency fluctuation (ALFF) and fractional ALFF (fALFF). The ALFF measures the signal intensity in low-frequency oscillations (LFOs) of local spontaneous neural activity of the brain [34] and has been proven to exhibit outstanding test-retest reliability [35]. Previous studies have investigated spontaneous neural activities in the GM and found decreased ALFF in patients with TIA [11, 36], providing evidence of brain dysfunction in TIA. Moreover, based on ALFF, fALFF was raised to characterize the relative contribution of a specific LFO to the whole frequency range, effectively reducing physiological noise and suppressing artifacts in nonspecific brain regions [35, 37]. According to previous studies, the ALFF method has been used to explore WM functional abnormalities in various diseases, such as Parkinson's disease (PD) [38], autism spectrum disorder (ASD) [2], and schizophrenia [39]. Although the fALFF method has not been used in conjunction with the ALFF method to assess the neural activity in the low-frequency range of WM, it is suggested that the combination of these two metrics can help obtain detailed information about brain activity in the low-frequency range than using individual method alone [40–42].

Machine learning algorithms have been widely used for diagnosing neuropsychiatric diseases and are powerful tools for classifying patients and healthy controls (HCs), which show great potential in clinical practice [43–47]. Among the numerous machine learning methods, the reinforcement learning approach is a promising method for addressing the diversity and complexity of the clinical conditions of the disease [48]. Learning through continuous trial-and-error in the interaction between agent and environment, reinforcement learning can adjust its actions according to the environmental feedback signal and arrive at the optimal decision [49–51]. In previous studies, reinforcement learning has been combined with rs-fMRI to recognize patients with early mild cognitive impairment (eMCI) by learning discriminative feature presentations from temporally embedded BOLD signals [52]. Based on probabilistic reinforcement learning tasks, it has been found that patients with treatment-resistant schizophrenia (TRS) and patients with non-treatment-resistant schizophrenia (NTR) can be separated by different neural mechanisms [53]. Additionally, when diagnosing myocarditis, an automatic classification model based on the deep reinforcement learning method can help effectively promote the automatic screening of noninvasive cardiac magnetic resonance (CMR) images [54]. In summary, reinforcement learning can be combined with different methods to distinguish patients from healthy individuals. Hence, we applied the reinforcement learning method to examine whether WM functional abnormalities could effectively differentiate patients with TIA from HCs.

In this study, functional alterations in the WM of patients with TIA were explored using two resting-state metrics (ALFF and fALFF) to determine whether there was WM functional damage in patients with TIA. Furthermore, a reinforcement learning approach was adopted to investigate whether the altered WM function of ALFF and fALFF could serve as effective neuromarkers for identifying patients with TIA.

## 2. Materials and Methods

### 2.1. Participants

Data were acquired from 51 patients with suspected TIA in the Department of Neurology at the Anshan Changda Hospital, Liaoning, China. Patients with transient neurological symptoms may have a vascular etiology, according to the assessment of clinical psychiatrists [11, 36]. Blood pressure, clinical features, symptom duration, and history of diabetes symptoms were assessed for each patient. In addition, ABCD2 scores (a simple score to identify individuals at high early risk of stroke after a TIA) were generated for each patient's risk of secondary stroke [55]. All patients underwent electrocardiography (ECG), carotid duplex ultrasound (CDU), and magnetic resonance imaging (MRI). The information of each patient was recorded as follows: history of TIA and stroke; current smoking and drinking behavior; previous risk factors such as hypertension, diabetes, and coronary artery disease [56]; medications used before MRI scan [57]; in-hospital assessment of arterial stenosis on CDU and magnetic resonance angiography (MR angiography); atrial fibrillation on ECG; brain infarction on diffusion-weighted imaging (DWI) and T2 fluid-attenuated inversion recovery (T2-FLAIR) [58]; and 1-year telephone follow-up for stroke and/or TIA episodes [59]. Participants with migraine, epilepsy, hemorrhage, leukoaraiosis, or psychiatric history were excluded from this study [60].

The 41 HCs matched for age and sex to the TIA group were recruited through an advertising campaign. None of the HCs had a history of physical illnesses, psychiatric disorders, or neurological disorders. This study was approved by the Ethics Committee of the Center for Cognition and Brain Disorders, Hangzhou Normal University. All participants provided written informed consent.

### 2.2. Physiological and Biochemical Tests

All participants underwent a series of physiological and biochemical tests within 24 h before scanning, which included systolic blood pressure, diastolic blood pressure, blood sugar level, total cholesterol, triglycerides, high-density lipoprotein cholesterol (HDL-C), and low-density lipoprotein cholesterol (LDL-C).

### 2.3. Data Acquisition

Neuroimaging data were acquired using a GE MR-750 3.0 T scanner (GE Medical Systems, Inc., Waukesha, WI, United States). The parameters for acquiring 3D high resolution T1-weighted anatomical images were as follows: time of repetition (TR) = 8100 ms, time of echo (TE) = 3.1 ms, matrix size = 256 × 256, voxel size = 1 mm × 1 mm × 1 mm, thickness/gap = 1/0 mm, field of view (FOV) = 256 mm^2^, and scanning time = 5 min. Gradient echo-planar imaging (EPI) images were captured with TR = 2000 ms, TE = 30 ms, flip angle (FA) = 60°, matrix size = 64 × 64, thickness/gap = 3.2/0 mm, slices = 43, and scanning time = 8 min. During resting-state fMRI scanning, all participants were required to remain still with their eyes closed, remain awake, and not think of anything systematically. All participants reported that they were not asleep during the scanning. The interval between the latest TIA attack time of patients with TIA and the MRI scan time was 6 hours–16 days.

### 2.4. Data Preprocessing

**Figure 1:**
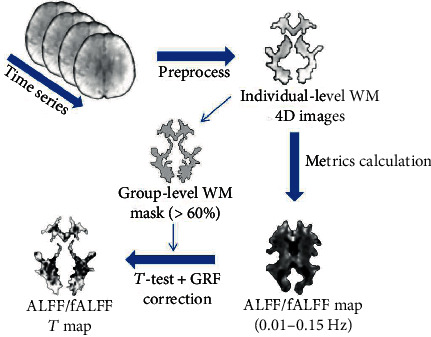
Flow chart of preprocessing, metric calculation, and statistical analysis in this study.

Preprocessing of rs-fMRI data was performed using SPM12 (http://www.fil.ion.ucl.ac.uk/spm) and RESTplus v1.24 [61] (http://www.restfmri.net/forum/REST) on Matlab 2017b (https://ww2.mathworks.cn/products/matlab.html), which consists of the following steps: (1) removal of the first 10 time points to stabilize magnetization and allow participants to acclimate to the scanning environment, keeping the remaining 230 volumes for further analysis. (2) Slice-time correction to adjust the data scanned simultaneously. (3) Realignment to correct slight head movements during scanning. (4) T1 image segmentation. The T1 images were coregistered with functional images and then segmented into GM, WM, and cerebrospinal fluid (CSF) using the New Segment algorithm [62]. (5) Removal of the linear trend to correct the signal drift. (6) Regression of the noise signals. To avoid eliminating signals of interest, we regressed only head motion (Friston-24 motion parameters [63]) and mean CSF, leaving WM and global signal out [28]. (7) Temporal scrubbing to censor the data at the spike without changing the correlation values by using the motion “spike” as a separate regressor [64, 65]. (8) Spatial smoothing (FWHM = 4 mm) was performed on the WM and GM images separately of each subject, as suggested in previous studies [28, 31]. (9) Normalization to the standard EPI template and resampling to 3 mm^3^ voxels using the DARTEL algorithm. (10) Extraction of individual-level WM 4D images. For each participant, we defined each voxel as GM, WM, and CSF based on its maximum probability from the T1 image segmentation results. This resulted in the individual-level WM 4D images. (11) Creation of group-level WM masks based on individual-level WM 4D images for follow-up statistical analysis. Voxels identified as WM in >60% of participants were adopted to create the WM mask [28]. The subcortical regions were then removed from the WM mask based on the Harvard–Oxford Atlas. The WM mask was also coregistered to the functional space and resampled to process the functional image [66]. A flowchart of the study is presented in Figure 1.

### 2.5. Metric Calculation

Metric calculations were conducted using the RESTplus software [61]. To avoid the mixture of WM and GM signals and reduce the interference of other noises on WM signals as much as possible, all calculations of these metrics were conducted on individual-level WM 4D images (Figure 1).

#### 2.5.1. ALFF Calculation

ALFF was calculated based on a fast Fourier transform (FFT). The time series of each voxel was transformed into the frequency domain, and the power spectrum was obtained. The square root of each power spectrum frequency was then calculated, and the mean square root was obtained for each voxel. Notably, according to Peer et al.'s research, the energy distribution in the frequency domain differs between WM and GM [28]. Additionally, previous studies demonstrated that 0.15 Hz was the highest expected frequency of hemodynamic signals generated by neurons [67, 68]. Therefore, to reduce the contributions of nonneuronal on BOLD fluctuations, the mean square root was calculated in the frequency band of 0.01–0.15 Hz [2, 16, 29, 30, 69]. Finally, the ALFF value for each voxel was divided by the average ALFF value (mALFF). In addition, the results of different frequency bands of 0.01–0.08 Hz [39], 0.01–0.10 Hz [24], and 0.01–0.15 Hz were compared in the case of other parameters that remained constant. The results are provided in detail in Supplementary Materials (Figure S1).

#### 2.5.2. fALFF Calculation

As for fALFF, identical to ALFF, 0.01–0.15 Hz was chosen as the frequency band. The ratio of the amplitude in the low-frequency range to the total amplitude in the entire frequency range (0–0.25 Hz) was calculated, representing the relative contribution of the oscillations in the low-frequency range to the signal variation in the entire frequency range. Finally, *z*-transformation was performed on the ALFF and fALFF maps of each participant. The comparison of different frequency bands is shown in the Supplementary Materials (Figure S2).

### 2.6. Statistical Analysis

Statistical analysis was performed using the Statistical Package for Social Sciences (SPSS) 26 (IBM Corp., Armonk, N.Y., USA) to examine the differences in demographic and clinical characteristics between patients with TIA and HCs. Age and clinical/physiological/biochemical characteristics were compared between the two groups using Student's *t*-test, and sex differences were compared using Pearson's chi-squared test. To examine the differences in neural activities in low-frequency bands of WM in patients with TIA and HCs, statistical significance was assessed at voxel-level *P* < 0.05 and cluster-level *P* < 0.05, corrected by Gaussian random fields (GRF) using RESTplus software [61]. In addition, a more rigorous threshold (voxel-level *P* < 0.01, GRF correction) was used to examine the results of the metric calculation and reinforcement learning (see Supplementary Materials Figure S3-S5). Considering the rigor of the statistical analysis, the group-level mask (> 60%) obtained in the preprocessing stage was used for statistical analysis to reduce the interference of non-WM signals. To support the future meta-analysis, we shared the original uncorrected *t*-maps (http://www.restfmri.net/TIA.tar). Finally, Pearson's correlation analysis was conducted to determine the correlation between resting-state metrics and clinical/physiological/biochemical characteristics. Specifically, the ALFF and fALFF values in WM regions showing group differences between the two groups were extracted and correlated with systolic blood pressure, diastolic blood pressure, blood sugar level, total cholesterol, triglycerides, HDL-C, and LDL-C. Statistical significance was set at *P* < 0.05.

### 2.7. Feature Extraction and *Q*-Learning Model Training

To evaluate whether alterations in ALFF and fALFF could serve as potential neuromarkers to distinguish patients with TIA from HCs, we performed a reinforcement learning analysis using the *Q*-learning algorithm [70, 71]. The steps were as follows: (1) the mean ALFF and fALFF values in WM regions showing significant differences between the two groups were used together to serve as features and were normalized from -1 to 1. According to previous studies on support vector machines (SVM), the combination of features of multiple metrics has a better classification effect than using single metric as the feature [36, 72–74]. (2) The parametric *Q*-learning method [75] was used to train the approximate *Q* value function with the linear model and obtain reward feedback by interacting with the environment to find the optimal *Q* value function and obtain the final classification. In this study, the discount factor *γ* was 0.9, and the learning rate *α* was 0.001. Finally, leave-one-out cross-validation (LOOCV) was performed to conduct cross-validation, which could help prevent overfitting [76, 77]. (3) The process described above was applied to each participant to evaluate the overall accuracy of parametric *Q*-learning. Accuracy, sensitivity, and specificity have been reported to quantify the performance of classification methods. The results of using the ALFF and fALFF features are presented in the Supplementary Materials (Figure S6-S7).

## 3. Results

### 3.1. Clinical Data

The final sample size was 89 participants (TIA, *n* = 48; HCs, *n* = 41). Three patients were excluded from further analysis owing to the unsatisfactory quality of multimodal MRI data, including incomplete coverage of the whole brain in the rs-fMRI scan and missing 3D T1 images. Of the 48 patients with TIA, 25 experienced TIA (not a first-time attack), 4 experienced a stroke, and 23 experienced the first episode. Detailed demographic and clinical information of all participants are summarized in Table 1.

As shown in Table 1, the TIA and HC groups were matched for age (*P* = 0.182) and gender (*P* = 0.640). Systolic blood pressure (*P* < 0.001), diastolic blood pressure (*P* = 0.007), blood sugar level (*P* = 0.001), total cholesterol (*P* = 0.045), and LDL-C (*P* = 0.004) were significantly higher in patients with TIA compared to HCs. The median ABCD2 score of patients with TIA was 4.

### 3.2. Between-Group Differences Results

**Figure 2:**
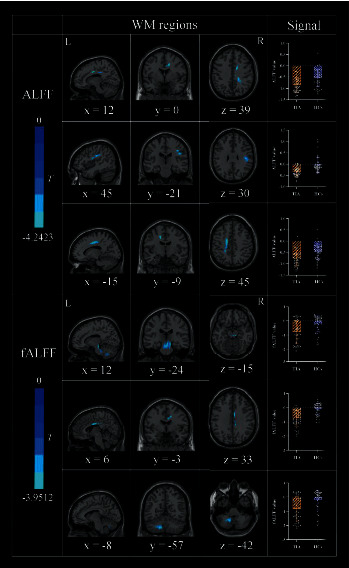
Regions of WM showing group differences in ALFF and fALFF together with signal values extracted from these regions.

Brain regions showing differences between groups in the metric analysis were reported based on the ICBM-DTI-81 white-matter label atlas (JHU DTI-based WM atlases, provided by Dr. Susumu Mori, Laboratory of Brain Anatomical MRI, Johns Hopkins University [78, 79]). For the ALFF calculations, patients with TIA showed decreased ALFF in the right cingulate gyrus, right superior longitudinal fasciculus, and left superior corona radiata compared with HCs (Table 2, Figure 2). The right cerebral peduncle, right cingulate gyrus, and middle cerebellar peduncle showed decreased fALFF in patients with TIA (Table 2, Figure 2). Among these brain regions, one cluster with a significant fALFF difference between patients with TIA and HCs was not reported in Table 2 due to it being off the JHU atlas.

### 3.3. Correlation Analysis

The ALFF and fALFF values were extracted from WM regions that showed significant differences between patients with TIA and HCs, and correlation analyses between these values and clinical/physiological/biochemical characteristics were conducted. There were no significant differences between the ALFF values in WM regions showing group difference and clinical measurements (*P* > 0.05). There was a significant negative correlation between the fALFF values extracted from the right cerebral peduncle and diastolic blood pressure (*r* = −0.316, *P* = 0.029), and a significant positive correlation between the fALFF values in the right cerebral peduncle and triglycerides (*r* = 0.310, *P* = 0.032). In addition, the fALFF values in the middle cerebellar peduncle showed a significant negative correlation with diastolic blood pressure (*r* = −0.320, *P* = 0.027) and a significant positive correlation with triglycerides (*r* = 0.327, *P* = 0.023).

### 3.4. Classification Results

**Figure 3:**
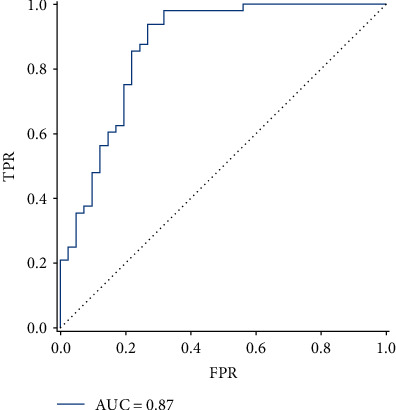
The receiver operating characteristic (ROC) curve of metrics. The image of ROC was displayed using the Matplotlib toolkit in Python. FPR, false positivity rate; TPR, true positivity rate; AUC, area under the ROC curve.

Accuracy, sensitivity, specificity, and precision were calculated to evaluate the classification ability of the parametric *Q*-learning model. The classifier achieved a total accuracy of 82.02%, sensitivity of 85.42%, specificity of 78.05%, precision of 82.00%, and area under the curve (AUC) of 0.87. The receiver operating characteristic (ROC) curve of the classifier is shown in Figure 3.

## 4. Discussion

In this study, two resting-state methods (ALFF and fALFF) were used for the first time to identify abnormalities in the low-frequency range of WM regions in patients with TIA. Moreover, the *Q*-learning algorithm of the reinforcement method was applied to detect neuromarkers that could be used to classify patients with TIA and HCs based on neuroimaging data. Additionally, we explored the relationship between functional abnormalities in WM and the clinical/physiological/biochemical features in patients with TIA. The results showed decreased ALFF in the right cingulate gyrus, right superior longitudinal fasciculus, and left superior corona radiata and decreased fALFF in the right cerebral peduncle, right cingulate gyrus, and middle cerebellar peduncle in patients with TIA. These findings suggest that resting-state metrics can effectively help explore low-frequency BOLD fluctuations in WM in patients with TIA and that these regions of WM showing decreased ALFF and fALFF might indicate that patients with TIA may develop motor and cognitive impairment and emotional problems. Moreover, the *Q*-learning algorithm provided sensitive information for classifying patients with TIA and HCs. These findings may help us gain a deeper understanding of the pathological mechanisms underlying TIA from the perspective of WM dysfunction.

The ALFF is considered a reliable method for detecting the intensity of spontaneous fluctuations and presenting spontaneous brain activity [34]. Recently, ALFF has been demonstrated to effectively reflect functional alterations in WM [32, 38], providing a new perspective for studying WM dysfunction in various diseases. In the present study, we found a decreased ALFF in the right cingulate gyrus, right superior longitudinal fasciculus, and left superior corona radiata in the WM region of patients with TIA. The cingulate gyrus is structurally complex and performs a wide range of functions. Noninvasive imaging has shown that the cingulate gyrus is associated with executive control, emotion, and pain [80]. Damage to the cingulate gyrus may lead to cognitive abnormalities in attention, memory, and emotional processing [81]. Based on prior research and the results of this study, we speculated that patients with TIA may show negative changes in cognitive functions, such as emotion, memory, and executive control. Anatomical studies have shown that the superior longitudinal fasciculus is the biggest associative fiber bundle system in the brain and is connected to the superior frontal gyrus and supplementary motor areas [82]. Patients with TIA are known to exhibit significant cognitive impairment compared with HCs, and the superior longitudinal fasciculus also plays a critical role in a wide range of cognitive functions [19, 83], which might indicate that the onset of TIA may lead to functional impairment in the WM region and affect the patient's cognitive function. The corona radiata consists of numerous tracts involving subcortical motor pathways, which is one of the most prominent motor-related neural fibers [84]. Jiang et al. [85] also found that when an ischemic stroke lesion is located in the corona radiata, it may interfere with the functional circuitry between the brainstem and frontal cortex, thereby interfering with the patient's emotional expression [86]. We speculated that the decrease in ALFF in the corona radiata may suggest the possibility of developing poststroke depression after a future stroke episode in patients with TIA.

fALFF is calculated as the ratio of the low-frequency range power spectrum to the entire frequency range power spectrum, which is effective in suppressing physiological noise compared to ALFF [35, 87]. To obtain more comprehensive information about low-frequency BOLD fluctuations in WM, we further investigated functional WM abnormalities in patients with TIA using the fALFF method, which is more sensitive to spontaneous neural activity [35]. This study found decreased fALFF in the right cerebral peduncle, right cingulate gyrus, and middle cerebellar peduncle in patients with TIA compared with HCs. Recent diffusion tensor imaging (DTI) studies of cerebral hemorrhage have shown that decreased fractional anisotropy (FA) values in the cerebral peduncle away from the hematoma region indicate neurodegenerative lesions [88, 89]. Koyama et al. [84] also demonstrated that changes in this region are viable predictors of motor outcome. Consistent with the ALFF findings, we also found decreased fALFF in the right cingulate gyrus of patients with TIA, suggesting the possibility of developing cognitive abnormalities in the future. Bilateral middle cerebellar peduncle lesions are commonly associated with cerebrovascular diseases [90]. S.H. Kim and J.S. Kim [91] found unilateral middle cerebellar peduncle lesions in acute stroke patients that manifested clinically as ocular motor abnormalities. Additionally, Zhou et al. [92] found that patients with bilateral middle cerebellar peduncle infarction developed ataxia, characterized by impaired motor coordination. Combined with insights from previous studies, we found that these WM regions with decreased fALFF in patients with TIA were all related to motor function. Hence, we speculated that patients with TIA and decreased fALFF in these WM regions may be at risk of impaired motor coordination and movement disorders (such as local movement disorders) in the future. Our results may shed new light on abnormal changes in these WM regions after the onset of TIA.

In the present study, we noted some variations between the results of the ALFF and fALFF analyses, except for the right cingulate gyrus, which exhibited a decrease in both the ALFF and fALFF. The ALFF reflects the power in the effective frequency range [34], and the fALFF measures the relative spontaneous neural activity in the effective frequency range over the entire frequency range [35]. Owing to the difference in the calculation methods of the two metrics, our results showed slight differences. In addition, the BOLD signal in fMRI reflects the activation of neurons and global physiological fluctuations [93], which may influence the estimation of ALFF. The fALFF is a modified index of ALFF that, relative to the ALFF, can improve the sensitivity and specificity to spontaneous neural activities [87, 94], may provide us with more sensitive information about low-frequency BOLD fluctuations in WM, and validate abnormal functional neural activity in patients with TIA. However, it is noteworthy that the results of ALFF and fALFF analyses both showed decreased low-frequency neural activities in WM of patients with TIA compared with HCs, which may suggest an impaired function in these motor, emotional, and cognitive-related WM regions caused by TIA onset. Combining these two metrics provided us with more comprehensive information on the neural activity in the low-frequency range in WM than using only one method, which also helped examine WM dysfunction in patients with TIA.

Correlation analysis revealed no significant correlations between the ALFF values extracted from WM regions showing group differences and clinical characteristics. In contrast, significant correlations were observed between values in several WM regions showing group differences in fALFF and clinical characteristics. Values in the right cerebral peduncle and middle cerebellar peduncle were negatively correlated with diastolic blood pressure. Demographic information revealed that diastolic blood pressure was significantly higher in patients with TIA than in HCs. According to the World Health Organization Hypertension Guideline, diastolic blood pressure is a risk factor for cardiovascular disease; it is closely associated with mental stress, anxiety, and other emotions [95], which correspond to symptoms presented by some stroke patients since they have developed pathological emotional manifestations such as depression, anxiety disorder, apathy, and psychosis after onset [96, 97]. In this study, we speculated that decreased fALFF values in the right cerebral peduncle and middle cerebellar peduncle may be associated with emotional challenges in patients with TIA. Triglycerides were previously considered a separate risk factor for ischemic stroke in elderly Chinese patients with hypertension [98]. Furthermore, high triglycerides are associated with pathophysiological processes and may contribute to an increased risk of ischemic stroke [99]. Our findings demonstrated that the fALFF values in the right cerebral peduncle and middle cerebellar peduncle of patients with TIA were positively correlated with triglyceride levels. These results imply that the two WM regions may serve as key predictors of ischemic stroke occurrence in the future.

Machine learning has been widely used in neuroscience and for diagnosing neuropsychiatric diseases and has shown good classification performance [100, 101]. More objective diagnostic criteria can be established through machine learning algorithms to help identify neuromarkers for TIA diagnoses. In this study, we combined two indicator features (ALFF and fALFF) and used the *Q*-learning algorithm to distinguish patients with TIA from HCs with an identification accuracy of 82.02% and satisfactory specificity, sensitivity, and precision, which helped establish diagnostic indicators. Therefore, the abnormal ALFF and fALFF values in the WM of the brain could be used as potential imaging biomarkers to differentiate patients with TIA from HCs. Furthermore, *Q*-learning is a promising method for studying these WM functional abnormalities.

## 5. Limitations

The present study had some limitations that should be interpreted with caution. First, the sample size of this study was comparatively small, and we would like to validate our results in the future with bigger sample size. Second, fMRI data of patients with TIA were not collected during the follow-up period, to the extent that we have not yet clarified how spontaneous activity in WM changes as TIA progresses. Future studies could be designed longitudinally to test whether current methods can be used to monitor disease progression. Finally, this study lacked information on the emotional condition of patients with TIA, such as depression and anxiety. Considering that our results showed that abnormal WM regions in patients with TIA might also have emotional problems; therefore, future studies can evaluate depression and anxiety in patients using appropriate scales to learn more about the relationship between WM neural activity and scale scores.

## 6. Conclusion

The present study demonstrated abnormal WM functional alterations in the low-frequency range in patients with TIA. Moreover, WM neural activity in the low-frequency range may serve as a potential neuromarker to differentiate patients with TIA from HCs. These findings provide novel insights into the underlying mechanisms in patients with TIA from the perspective of WM function. Abnormal WM regions may serve as the basis for the clinical diagnosis and prevention of stroke in patients with TIA.

## Figures and Tables

**Table 1:** Demographic and clinical information of all participants.

Variables	TIA (*n* = 48)	HCs (*n* = 41)	*P* value
Age (year, mean ± SD)	57.60 ± 9.78	55.02 ± 8.03	0.182*^t^*
Sex (male/female)	37/11	30/11	0.670^*χ*^
Systolic blood pressure (mmHg, mean ± SD)	145.54 ± 20.75	127.55 ± 19.53^a^	<0.001*^t^*
Diastolic blood pressure (mmHg, mean ± SD)	86.67 ± 10.38	80.03 ± 10.90^a^	0.007*^t^*
Blood sugar level (mmol/L, mean ± SD)	6.30 ± 2.11	5.12 ± 0.74^a^	0.001*^t^*
Total cholesterol (mmol/L, mean ± SD)	5.24 ± 1.14	4.75 ± 1.01^a^	0.045*^t^*
Triglycerides (mmol/L, mean ± SD)	1.60 ± 0.94	1.92 ± 1.35^a^	0.213*^t^*
HDL-C (mmol/L, mean ± SD)	1.11 ± 0.24	1.05 ± 0.29^a^	0.306*^t^*
LDL-C (mmol/L, mean ± SD)	3.31 ± 0.97	2.69 ± 0.90^a^	0.004*^t^*
ABCD2 scores (median)	4 (2–6)		
Smoking, no. (%)	31 (64.58%)		
Drinking, no. (%)	20 (41.67%)		
Hypertension, no. (%)	22 (45.83%)		
Diabetes, no. (%)	8 (16.67%)		
Coronary artery disease, no. (%)	2 (4.17%)		
Atrial fibrillation, no. (%)	1 (2.08%)		
Medication, no. (%)	—		
Antiplatelets, no. (%)	48 (100%)		
Statins, no. (%)	2 (4.17%)		
DWI hyperintensity, no. (%)	6 (12.50%)		
Vessel stenosis, no. (%)	9 (18.75%)		
TIA/stroke attack in one year follow-up, no. (%)	12 (27.27%)^b^		

Note: *^t^* The *P* value was obtained by Student's *t*-test; ^*χ*^ The *P* value was obtained by two-tailed Pearson chi-square *t*-test; ^a^ Data were missing for 6 controls; ^b^ Four patients dropped out in the one-year follow-up; ABCD2 is a simple score to identify individuals at high early-risk of stroke after a TIA. TIA: transient ischemic attack; HCs: healthy controls; HDL-C: high-density lipoprotein cholesterol; LDL-C: low-density lipoprotein cholesterol.

**Table 2:** Regions of WM showing abnormal ALFF and fALFF in patients with TIA compared with HCs.

Metrics	Tract (JHU-atlas)	Voxels	MNI coordinates			*T* value
			*x*	*y*	*z*	
ALFF	Cingulum_R	120	12	0	39	-3.7533
	Superior_longitudinal_fasciculus_R	119	45	-21	30	-4.2423
	Superior_corona_radiata_L	102	-15	-9	45	-3.7579
fALFF	Cerebral_peduncle_R	116	12	-24	-15	-3.7614
	Cingulum_R	109	6	-3	33	-3.9512
	Middle_cerebellar_peduncle	90	-18	-57	-42	-3.8369

Note: The statistical threshold was set at voxel with *P* < 0.05 and cluster with *P* < 0.05 for GRF correction. Cingulum_R: right cingulate gyrus; Superior_longitudinal_fasciculus_R: right superior longitudinal fasciculus; Superior_corona_radiata_L: left superior corona radiate; Cerebral_peduncle_R: right cerebral peduncle; Middle_cerebellar_peduncle: middle cerebellar peduncle; TIA: transient ischemic attack; HCs: healthy controls; MNI: Montreal Neurological Institute; ALFF: amplitude of low-frequency fluctuation; fALFF: fractional ALFF.

## Data Availability

The raw data supporting the conclusions of this study will be made available by the authors, without undue reservation.

## Supplementary Materials

Supplementary Materials Figure S1. Regions of WM showing group differences in ALFF in different frequency bands. Figure S2. Regions of WM showing group differences in fALFF in different frequency bands. Figure S3. Regions of WM showing group differences in ALFF with GRF correction using different thresholds (voxel-level *P* < 0.05 and *P* < 0.01). Figure S4. Regions of WM showing group differences in fALFF with GRF correction using different thresholds (voxel-level *P* < 0.05 and *P* < 0.01). Figure S5. The receiver operating characteristic (ROC) curve of metrics under different thresholds (voxel-level *P* < 0.05 and *P* < 0.01, GRF correction) served as features.^a^ By using the mean ALFF and fALFF values in the clusters showing significant differences (voxel-level *P* < 0.05, GRF correction) between the two groups as features, the classifier achieved a total accuracy of 82.02%, sensitivity of 85.42%, specificity of 78.05%, precision of 82.00%, and AUC of 0.87; ^b^ By using the mean ALFF and fALFF values in the clusters showing significant differences (voxel-level *P* < 0.01, GRF correction) between the two groups as features, the classifier achieved a total accuracy of 80.90%, sensitivity of 77.08%, specificity of 85.37%, precision of 86.05%, and AUC of 0.77. The image of ROC was displayed using the Matplotlib toolkit in Python. FPR, false positivity rate; TPR, true positivity rate; AUC, area under the ROC curve. Figure S6. The receiver operating characteristic (ROC) curve of metrics (ALFF). By using the mean ALFF values in the clusters showing significant differences (voxel-level *P* < 0.05, GRF correction) between the two groups as features, the classifier achieved a total accuracy of 78.65%, sensitivity of 75.00%, specificity of 82.93%, precision of 83.72%, and AUC of 0.77. The image of ROC was displayed using the Matplotlib toolkit in Python. FPR, false positivity rate; TPR, true positivity rate; AUC, area under the ROC curve. Figure S7. The receiver operating characteristic (ROC) curve of metric (fALFF). By using the mean fALFF values in the clusters showing significant differences (voxel-level *P* < 0.05, GRF correction) between the two groups as features, the classifier achieved a total accuracy of 70.79%, sensitivity of 79.17%, specificity of 60.98%, precision of 70.37%, and AUC of 0.75. The image of ROC was displayed using the Matplotlib toolkit in Python. FPR, false positivity rate; TPR, true positivity rate; AUC, area under the ROC curve.
