# Supplementary material for: The White Matter Functional Abnormalities in Patients with Transient Ischemic Attack: A Reinforcement Learning Approach
Source: Neural Plast. 2022 Oct 17;2022:1478048. doi: 10.1155/2022/1478048 (PMC9592236; doi:10.1155/2022/1478048)
Supplement: Supplementary Materials — Figure S1. Regions of WM showing group differences in ALFF in different frequency bands. Figure S2. Regions of WM showing group differences in fALFF in different frequency bands. Figure S3. Regions of WM showing group differences in ALFF with GRF correction using different thresholds (voxel-level P < 0.05 and P < 0.01). Figure S4. Regions of WM showing group differences in fALFF with GRF correction using different thresholds (voxel-level P < 0.05 and P < 0.01). Figure S5. The receiver operating characteristic (ROC) curve of metrics under different thresholds (voxel-level P < 0.05 and P < 0.01, GRF correction) served as features.a By using the mean ALFF and fALFF values in the clusters showing significant differences (voxel-level P < 0.05, GRF correction) between the two groups as features, the classifier achieved a total accuracy of 82.02%, sensitivity of 85.42%, specificity of 78.05%, precision of 82.00%, and AUC of 0.87; b By using the mean ALFF and fALFF values in the clusters showing significant differences (voxel-level P < 0.01, GRF correction) between the two groups as features, the classifier achieved a total accuracy of 80.90%, sensitivity of 77.08%, specificity of 85.37%, precision of 86.05%, and AUC of 0.77. The image of ROC was displayed using the Matplotlib toolkit in Python. FPR, false positivity rate; TPR, true positivity rate; AUC, area under the ROC curve. Figure S6. The receiver operating characteristic (ROC) curve of metrics (ALFF). By using the mean ALFF values in the clusters showing significant differences (voxel-level P < 0.05, GRF correction) between the two groups as features, the classifier achieved a total accuracy of 78.65%, sensitivity of 75.00%, specificity of 82.93%, precision of 83.72%, and AUC of 0.77. The image of ROC was displayed using the Matplotlib toolkit in Python. FPR, false positivity rate; TPR, true positivity rate; AUC, area under the ROC curve. Figure S7. The receiver operating characteristic (ROC) curve of metric (fALFF). [file 1478048.f1.docx]

**Supplementary Materials**

**
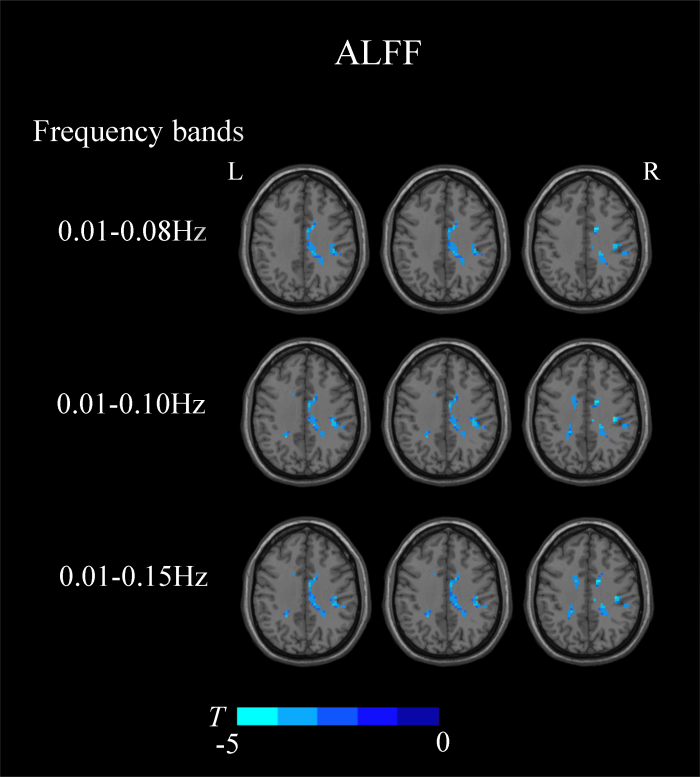
**

**Figure S1.** Regions of WM showing group differences in ALFF in different frequency bands.

**
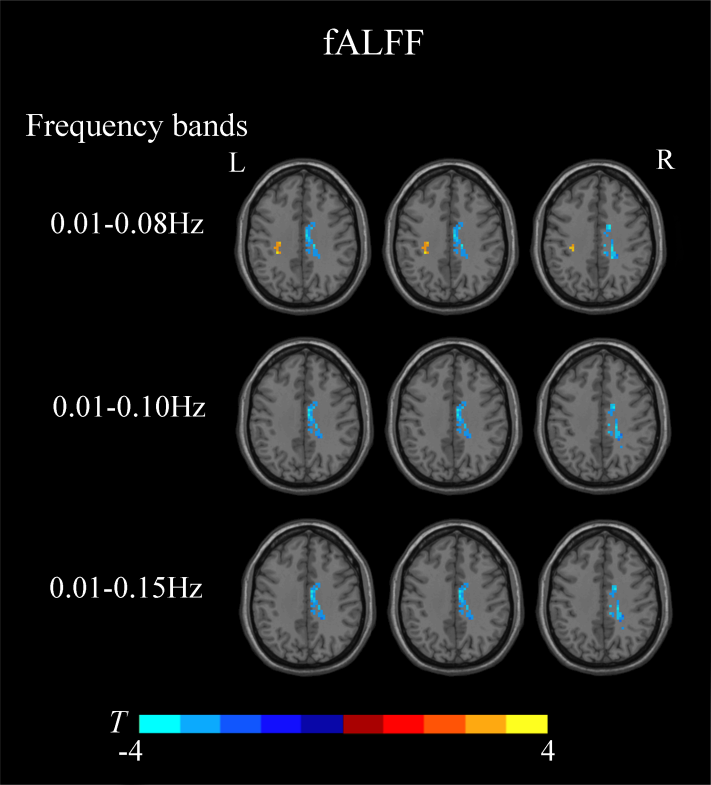
**

**Figure S2.** Regions of WM showing group differences in fALFF in different frequency bands.

**
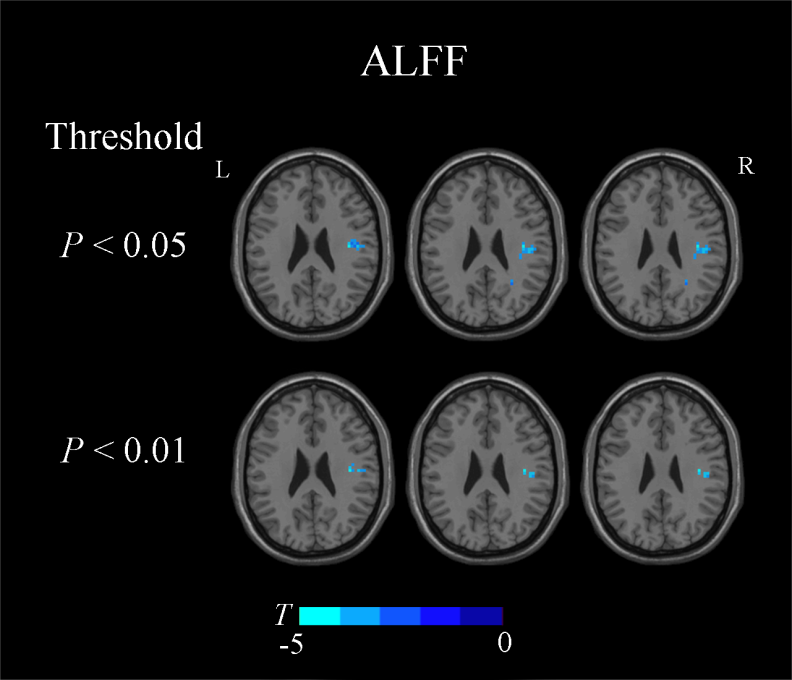
**

**Figure S3.** Regions of WM showing group differences in ALFF with GRF correction using different thresholds (voxel-level *P* < 0.05 and *P* < 0.01).

**
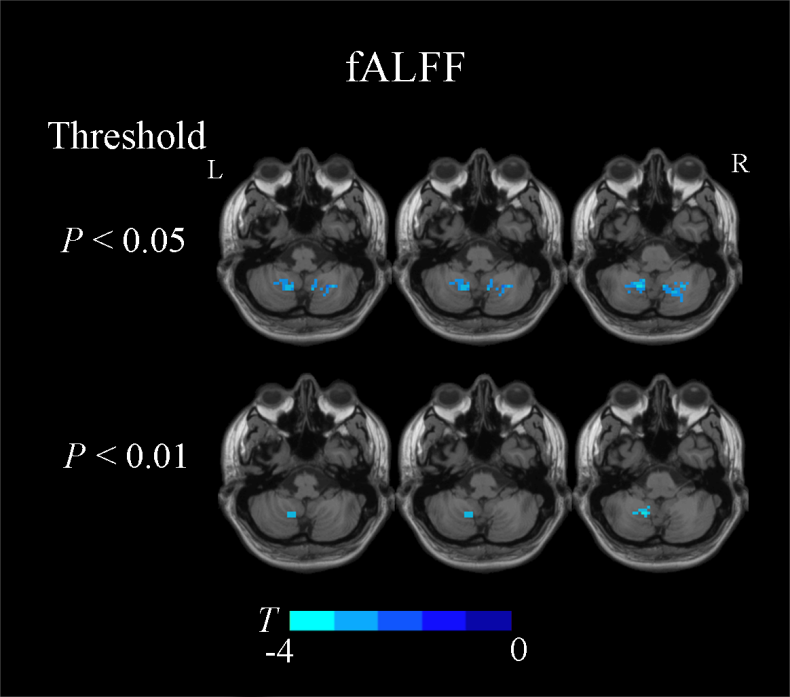
**

**Figure S4.** Regions of WM showing group differences in fALFF with GRF correction using different thresholds (voxel-level *P* < 0.05 and *P* < 0.01).

**
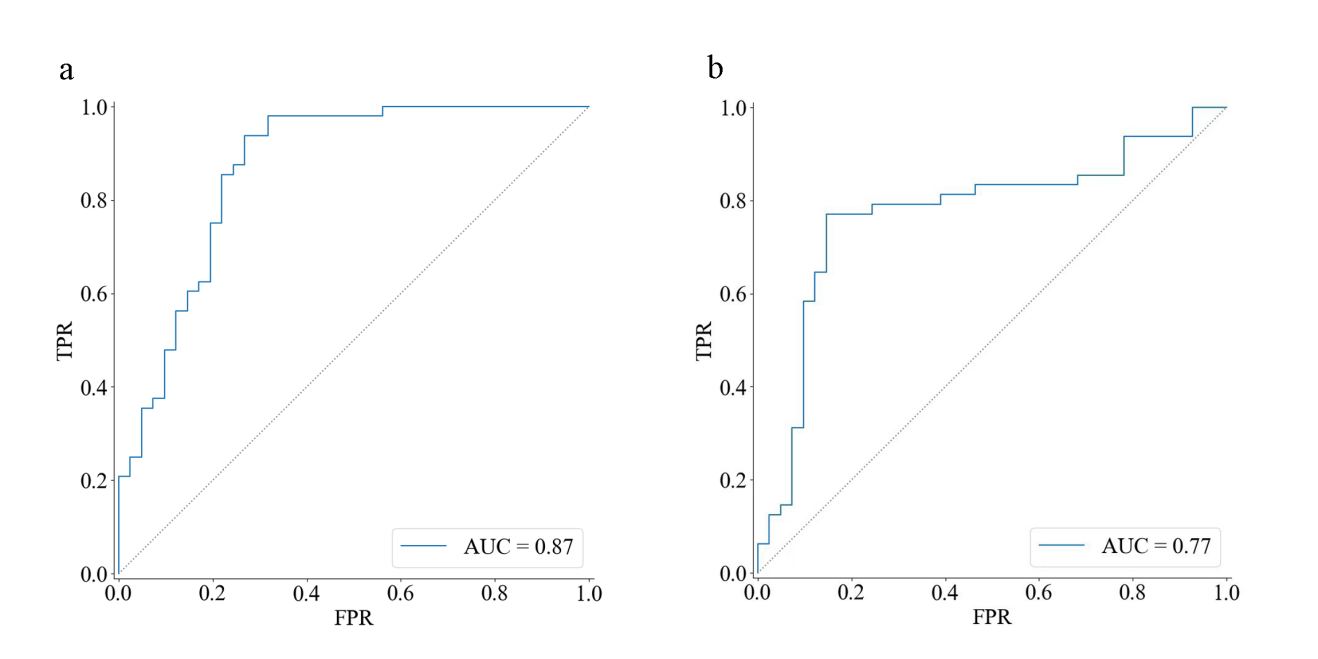
**

**Figure S5.** The receiver operating characteristic (ROC) curve of metrics under different thresholds (voxel-level *P* < 0.05 and *P* < 0.01, GRF correction) served as features. ^a^ By using the mean ALFF and fALFF values in the clusters showing significant differences (voxel-level *P* < 0.05, GRF correction) between two groups as features, the classifier achieved a total accuracy of 82.02%, sensitivity of 85.42%, specificity of 78.05%, precision of 82.00%, and AUC of 0.87; ^b^ By using the mean ALFF and fALFF values in the clusters showing significant differences (voxel-level *P* < 0.01, GRF correction) between two groups as features, the classifier achieved a total accuracy of 80.90%, sensitivity of 77.08%, specificity of 85.37%, precision of 86.05%, and AUC of 0.77. The image of ROC was displayed using the Matplotlib toolkit in Python. *FPR*, false positivity rate; *TPR*, true positivity rate; *AUC*, area under the ROC curve.

**
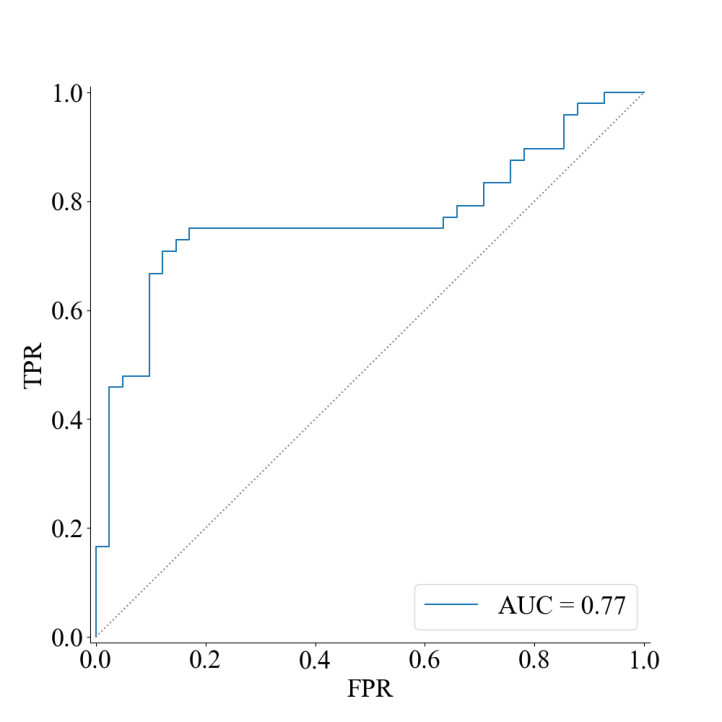
**

**Figure S6.** The receiver operating characteristic (ROC) curve of metrics (ALFF). By using the mean ALFF values in the clusters showing significant differences (voxel-level *P* < 0.05, GRF correction) between two groups as features, the classifier achieved a total accuracy of 78.65%, sensitivity of 75.00%, specificity of 82.93%, precision of 83.72%, and AUC of 0.77. The image of ROC was displayed using the Matplotlib toolkit in Python. *FPR*, false positivity rate; *TPR*, true positivity rate; *AUC*, area under the ROC curve.


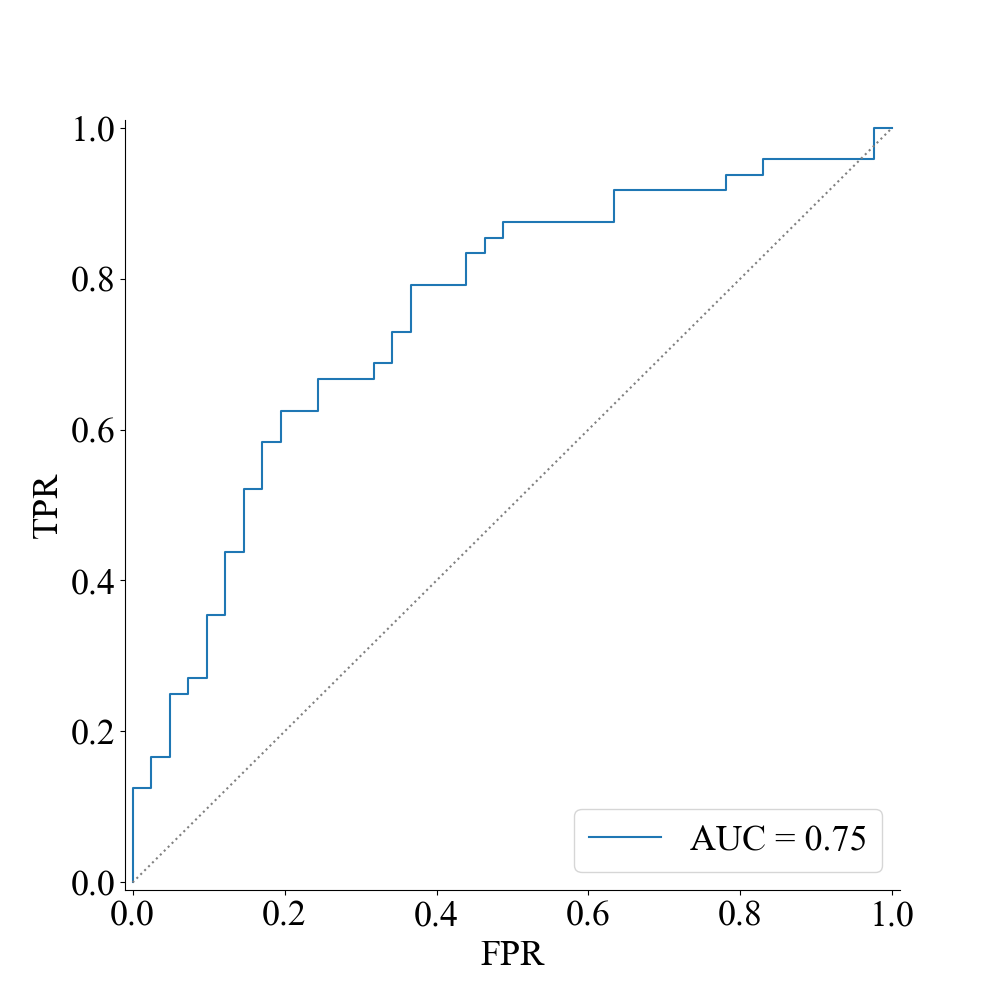


**Figure S7.** The receiver operating characteristic (ROC) curve of metric (fALFF). By using the mean fALFF values in the clusters showing significant differences (voxel-level *P* < 0.05, GRF correction) between two groups as features, the classifier achieved a total accuracy of 70.79%, sensitivity of 79.17%, specificity of 60.98%, precision of 70.37%, and AUC of 0.75. The image of ROC was displayed using the Matplotlib toolkit in Python. *FPR*, false positivity rate; *TPR*, true positivity rate; *AUC*, area under the ROC curve.
